# Short stem total hip arthroplasty for osteonecrosis of the femoral head in patients 60 years or younger: a 3- to 10-year follow-up study

**DOI:** 10.1186/s12891-017-1662-6

**Published:** 2017-07-17

**Authors:** Antonio Capone, Fabrizio Bienati, Stefania Torchia, Daniele Podda, Giuseppe Marongiu

**Affiliations:** 1 Orthopaedic Clinic of the Department of Surgical Science, University of Cagliari, Ospedale Marino, Lungomare Poetto 12, 09126 Cagliari, Italy; 2 Trauma and Orthopaedic Department, Ospedale Santissima Trinità, Via Is Mirrionis 92, 09121 Cagliari, Italy

**Keywords:** Osteonecrosis of the femoral head, Hip arthroplasty, Short stems, Ceramic on ceramic bearings

## Abstract

**Background:**

In young patients with osteonecrosis of the femoral head (ONFH), short-stem total hip arthroplasty (THA) could allow a potential advantage in preserving metaphyseal bone-stock, when revision surgery might become necessary. However, only a few studies have evaluated the outcome of short-stem THAs in ONFH. We reviewed the prospectively collected data of a cementless partial neck-retaining short-stem with ceramic-on-ceramic bearings in ONFH patients.

**Methods:**

Thirty patients (37 hips) younger than 60 years (mean age at surgery, 51.5 years) underwent THA with the NANOS® stem (Smith and Nephew, Marl, Germany) from January 2006 to December 2012. All patients received a 32-mm or 36 mm ceramic femoral head. Harris hip score, WOMAC and UCLA activity score were recorded. Postoperative radiographs were evaluated for bone-implant fixation and osteolysis. Further analysis correlated clinical findings with implants characteristics and patient demographics at mean 5.6 years’ follow-up (range, 3–10 years).

**Results:**

The clinical and functional results improved significantly (*p* < 0.001). At latest follow-up, mean HHS, WOMAC, and UCLA activity scores were 90 (range, 71–100), 94 (range, 76–100), and 6.3 (range, 4–10) points, respectively. The diameter of the femoral head did not influence the clinical outcome (*p* = 0.661). All hips showed bone ingrowth fixation of the acetabular and femoral components. No patients showed osteolysis. No revision for any reason was performed during the study period.

**Conclusions:**

The excellent clinical results and fixation pattern at mean 5.6 years’ follow-up reveal this implant as a reliable option in advanced stage of ONFH either. Further investigations are crucial to determine the long-term durability and to assess whether the association of ceramic-on-ceramic bearings, can be useful to achieve longer survivorship and lower complications rates.

**Trial registration:**

Registry number: ISRCTN 91336248; date of registration: 04/07/2017.

## Background

Osteonecrosis of the femoral head (ONFH) is a complex syndrome in which a localized area of bone becomes necrotic, primarily due to an impairment of its blood supply [1]. With regard to non-traumatic ONFH, some of the most common and well-described risk factors include high dose corticosteroid use, chemotherapic and immunosuppressant agents, excessive alcohol consumption, and smoking [2–7]. As stated by Mont et al. [8, 9], if untreated ONFH unfortunately is often progressive. Once irreversible collapse of the articular surface has taken place, according to the existing literature, attempts to preserve the femoral head are less successful and better results are achieved through joint-replacing techniques [1, 10–12]. Common joint-replacement treatments include hip resurfacing, total hip arthroplasty (THA) and short stem THA [13–18]. High failure and complications rates have been reported for both hemi-resurfacing and total hip resurfacing [10, 13, 19, 20], therefore total hip resurfacing is now considered a valuable option only for restricted indications [21, 22].

Historically, traditional THAs performed on patients with ONFH were reported to have lower survivorship and worse outcomes when compared to THAs performed for other diagnoses, with a failure rate ranging from 39% to 53%, in first generation hip arthroplasty [23–25]. A systematic literature review, showed a significant decrease in revision rates in patients who had surgery in 1990 or later, versus those who underwent surgery before 1990, with revision surgery performed or indicated in 17% (0–50%) and 3% (range 0–7%) of hips respectively [26]. This trend was confirmed in recent reviews that showed survivorship higher than 90% and remarkable improvements in clinical outcomes of contemporary cementless THAs performed for ONFH, due to major advances in the production techniques of implants, in bearing coupling and improved polyethylene sterilization and storage [27, 28].

Even though according to the literature traditional stems showed to be a reliable option in ONFH, due to the younger age of patients, short-stem arthroplasty could allow a potential advantage in metaphyseal bone stock preserving, when revision surgery might become necessary [6, 29]. A number of different short-stem designs have been developed [12]. Unfortunately, there are only few studies presenting the clinical outcome of short-stem THAs in ONFH [16–18].

Based on these considerations, we reviewed the prospectively collected data of a partial neck-retaining short stem and ceramic-on-ceramic bearings in patients younger than 60 years with progressed ONFH. The aim of the study is to assess the clinical and radiological outcome at mid – term follow up.

## Methods

From January 2006 to December 2012, a partial neck-retaining cementless femoral short-stem was used for 39 THAs in 32 patients 60 years of age or younger, due to osteonecrosis of the femoral head; 7 patients (21.87%) had bilateral THAs. Patients were excluded from the study if they were older than 60 years or had a follow up of less than 3 years after the operation. Two patients were lost to follow-up in the interim, meaning that 30 patients (37 hips, 94.87%) were available for clinical and radiographic evaluation at a mean follow-up of 5.6 years (range, 3–10 years). During the study period, in our institution 24 THAs (20 patients) due to ONFH were performed by the senior author (AC) using other implants in patients older than 60 years of age. The study was approved by the Institution review board, and all patients provided written informed consent. The mean age of the patients at the time of the index arthroplasties was 51.5 years (range, 27–60 years). There were 31 men and 1 women. According to ONFH Steinberg Classification, 19 hips were Stage IV (51.35%) and 18 were Stage V (48.64%) [30]. The morphology of the proximal femur was Dorr [31] Type A in 21 hips (56.75%) and Type B in 16 hips (43.25%). The presumed cause of osteonecrosis was idiopathic osteonecrosis in 28 hips (75.67%), corticosteroid use for seronegative rheumatic disease in 5 (13.51%), pharmacological treatment for leukemia/lymphoma in 4 (10.81%) (Table 1).

**Table 1:** Demographic data of patients

Demographic	Number
Number of patients (hips)	30 (37)
Male:female	29:1
Mean age (years)	51.5 (27–61)
Right:left side	18:19
Diagnosis (hips): Osteonecrosis	
Idiopathic	28 (75.68%)
Seronegative rheumatic disease	5 (13.52%)
Leukemia-Limphoma	4 (10.80%)
Steinberg stage	
IV	19 (51.35%)
V	18 (48.65%)
Duration of follow-up (years)	5.6 (3–10)

Ranges or percentages in parentheses

All patients received a partial neck-retaining short-stem (NANOS®; Smith and Nephew, Marl, Germany). The implant consisted of a titanium alloy stem with a calcium-phosphate coating on approximately 75% of the stem (BONIT®; DOT GmbH, Germany). A 32- or 36-mm diameter ceramic femoral head (BIOLOX-forte; CeramTec, Plochingen, Germany) was implanted in 13 hips and 24 hips, respectively. A cementless porous-coated acetabular shell (EP-FIT PLUS™; Smith and Nephew, Marl, Germany), was used in all hips, ranging from 46 to 58 mm. A ceramic liner (BIOLOX-forte; CeramTec, Plochingen, Germany) was used in all hips. All procedures were performed by the senior author (AC) through a modified Hardinge approach, in supine position. The index operation was performed under epidural anesthesia in all 32 patients. After femoral head resection, at least 10 mm from the base of the great trochanter and perpendicular to the femoral neck, the femoral path was prepared with cancellous bone compactors. The stem was then inserted with a press-fit technique. In all cases, the acetabulum was reamed line-to-line or 1 mm more than the diameter of the component used. The patients were allowed to stand on the first postoperative day and progress to full weight-bearing with crutches. Patients were recommended to use a pair of crutches for 4 weeks. Clinical and radiographic follow-up was performed at 1 months, 3 months, 6 months, 1 year, and yearly thereafter. The Harris hip score (HHS) [32], the Western Ontario and McMaster Universities Osteoarthritis Index (WOMAC) [33] and the UCLA score [34] were determined before surgery and at each follow up examination. Patients were asked about thigh pain. The evidence of any clicking or squeaking sound emanating from the ceramic-on-ceramic bearing was recorded. Radiographs were analyzed by a research fellow (GM) who had no knowledge of the patient’s identity. An anterior-posterior radiograph of the pelvis with both hips in slight neutral rotation and no abduction was taken for every patient. A frog-leg lateral radiograph was also made of each hip. To measure the inclination of the acetabular component a line that joined the inferior margins of the two acetabular teardrops on the AP pelvic radiograph was drawn (inter-teardrop line), and then the angle of abduction was determined by the intersection line marked through the plane of opening of the socket. The level of neck osteotomy was considered correct when performed at least at 10 mm from the great trochanter. Stem position was considered varus or valgus when the tip of the stem slightly touched the medial or lateral cortical, respectively. The modification of the acetabular center of rotation was determined by radiographic measurements of the distance between the hip center of rotation and one horizontal and one vertical reference line [35]. The femoral offset measurements were performed by calculating the horizontal distance between the center of rotation and the femoral anatomical axis [36]. Leg length discrepancy (LLD) was investigated as the difference between the distances from the inter-teardrop line and the tip of the lesser trochanter of both hips. The stability of the acetabular component was determined according to Manley criteria [37]. Any site of acetabular osteolysis was recorded according to the system of DeLee and Charnley [38] and migration was assessed as described by Massin et al. [35]. Femoral stem fixation was investigated for bone ingrowth, stable fibrous fixation or unstable fibrous fixation according to Engh [39]. Subsidence was investigated as described previously by Kim [40]. Loosening of the femoral component was defined when there was a progressive axial of more than 3 mm or a varus or a valgus shift of more than 3° [40]. Osteolysis was defined as any radiolucency line at the bone-prosthesis interface according to the seven zones of Gruen [41]. Proximal femoral stress shielding and bone resorption was graded radiographically, as described by Engh et al. [42]. Heterotopic ossification, if present, was graded according to the classification of Brooker [43].

### Statistical analysis

The changes in clinical scores between pre-surgery and follow-up were evaluated using paired t-test. Analysis of covariance (ANCOVA) models including the pre-surgery values were used to assess the effect of Steinberg class (IV vs. V), diagnosis (idiopathic vs. secondary) and head size (32- vs. 36-mm) on the changes in clinical scores and radiographic data. For all analyses, a confidence interval level of 95% was selected and statistical significance has been set at *p* values of <0.001. Statistical analysis was performed using SAS Software Version 9.4. (SAS Institute Inc., Cary, NC, USA).

## Results

A statistically significant clinical and functional improvement was observed in HHS, WOMAC, and UCLA activity scores (Table 2).

**Table 2:** Clinical results

	Preoperative	Follow-up at 5.6 years	*p* value (Student’s two tailed paired test)
Harris hip score (points)	53 (range, 35–67)	90 (range, 71–100)	<0.001
Excellent (90–100)	0	34 (91.9%)	-
Good (80–89)	0	0	-
Fair (70–79)	0	3 (8.1%)	-
Poor (<70)	37 (100.0%)		-
WOMAC score (points)	53 (range, 40–67)	94 (range, 76–100)	<0.001
UCLA activity score (points)	2.9 (range, 2–4)	6.3 (range, 4–10)	<0.001
Thigh pain	-	None	-
Clicking Sound	-	1 (2.56%)	-
Squeaking sound	-	None	-

Values are expressed as mean, with range or percentages in parentheses

The preoperative UCLA activity score was 2.9 points (range, 2–4 points), which improved to 6.3 points (range, 4–10 points) at the final follow up. This improvement was statistically significant (*p* < 0.001). 23 patients (76.6%) referred that regularly participate in active events such as bicycling, bowling and 10 (30%) of these patients sometimes participate in impact sports such as jogging, tennis and skiing.

All patients were able to stop using the cane within 3 months. At the final follow-up (range 3–10 years), three patients had fair results at HHS. Two of these patients had a mild limp related to abductor mechanism deficiency. The other one developed ONFH of the non-operated hip. No patient complained thigh pain.

No statistically significant difference in the mean follow-up scores for HHS (*p* = 0.588), WOMAC (*p* = 0.104) and UCLA activity score (*p* = 0.753) was found between the idiopathic ONFH group and secondary ONFH group. The mean follow-up scores were similar for HHS (*p* = 0.747), WOMAC (*p* = 0.541), UCLA activity score (*p* = 0.787) in the Steinberg stage V group and in Steinberg stage IV group.

No statistically significant difference in the mean follow-up scores for HHS (*p* = 0.022), WOMAC (*p* = 0.661) and UCLA activity score (*p* = 0.363) was found between the 32-mm alumina head group and 36-mm group. No statistically significant difference in complication rates between the two groups was found (*p* = 0.567). One patient (2.70% of hips) in the 36-mm group had early dislocation that was successfully treated with closed reduction. An other patient (2.70% of hips) in the 36-mm group had clicking sounds without evidence of alumina head or liner fracture, and no squeaking was detected.

**Fig. 1:**
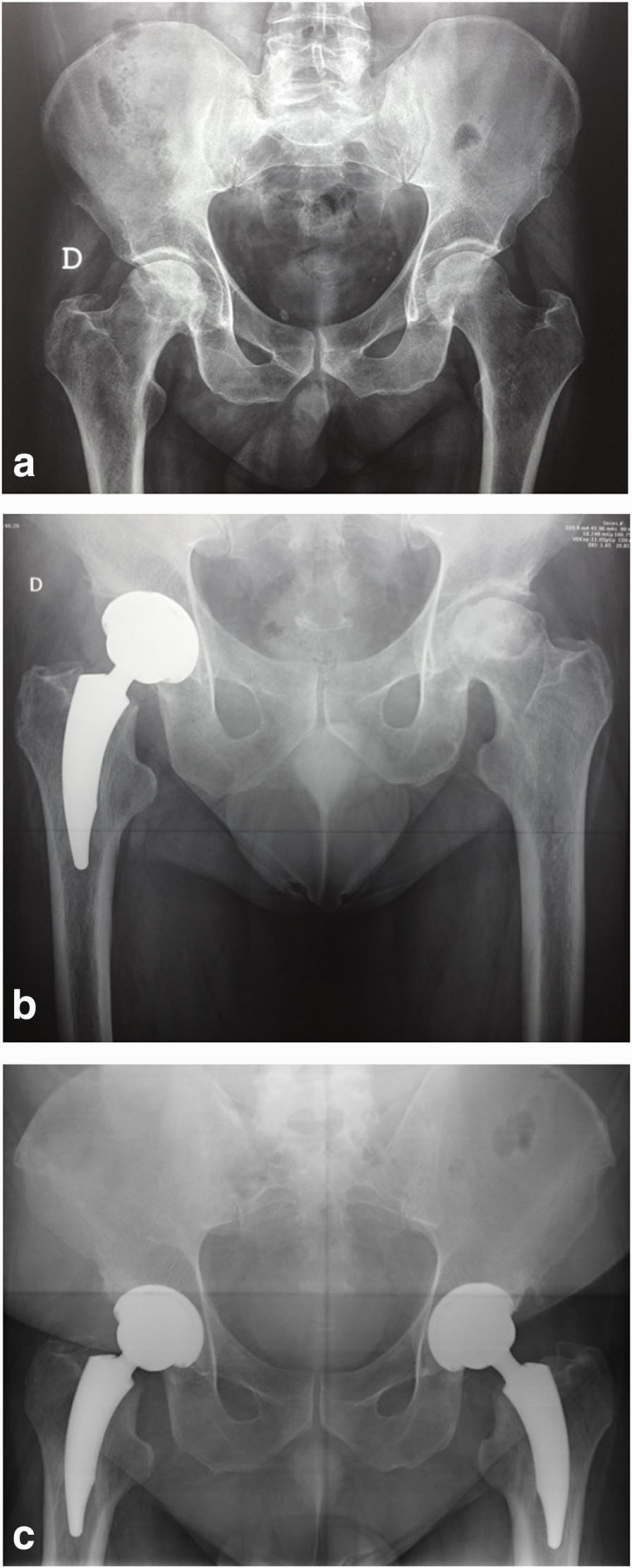
**a-c** Radiographs show the case of a 48-year-old male patient who had osteonecrosis of both femoral heads. **a** AP view of the pelvis before surgery shows Steinberg Stage IV osteonecrosis of the right femoral head. **b** AP view of the pelvis taken 1 years postoperatively reveals that acetabular and femoral components are well fixed in a satisfactory position; the left hip shows Steinberg Stage V osteonecrosis of the left femoral head. **c** At 7-year follow-up AP view Grade 1 calcar resorption is evident in both hips, without signs of stem loosening and osteolysis

At postoperative plain radiographs, stem position was neutral in 33 (89.18%) cases, valgus in 2 (5.40%) and varus in 2 (5.40%). The acetabular inclination between 40° to 50° (mean value 47°; range, 44°-52°) was obtained in 38 hips (97.43%). Osseointegration was complete for all hips at minimum 3-year follow-up, as confirmed by radiographic signs of fixation and the absence of stem migration. No hip had a subsidence of more than 3.0 mm or 3°shift in varus/valgus. 9 hips (24.32%) exhibited Grade 1 stress shielding in the calcar region and 1 (2.70%) had Grade 2. No acetabular or femoral osteolysis was identified in any hip. Grade 1 heterotopic ossification occurred in 5 hips (13.51%) (Table 3). No hip had revision or aseptic loosening at mean 5.6-years follow-up (range 3–10) (Fig. 1).

**Table 3:** Radiographic results

Parameter	Number
Dorr bone type	
A	21 hips (56.75%)
B	16 hips (43.25%)
Acetabular component position	
Inclination	47.0° (44° - 52°)
Femoral component position	
Neutral	33 hips (89.18%)
Valgus	2 hips (5.40%)
Varus	2 hips (5.40%)
Level of osteotomy (mm)	13.85 (10–20)
Center of rotation	
Horizontal (mm)	38 ± 4.5 (30–46)
Vertical (mm)	17.3 ± 4.2 (11–26)
Femoral offset (mm)	46 ± 4.7 (38–56)
Limb-length (mm)	37.4 ± 3.65 (28–45)
Radiolucent line (>1 mm)	0 hip (0.00%)
Migration of acetabular or femoral component	0 hip (0.00%)
Stress shielding	
Grade 1	9 hips (24.32%)
Grade 2	1 hip (2.70%)
No	27 hips (72.97%)
Osteolysis	0 hip (0.00%)
Heterotopic ossification	
Grade 1	5 hips (13.51%)
No	32 hips (86.49%)

Ranges or percentages in parentheses

## Discussion

Early reports on THAs showed unsatisfactory results in patients with ONFH [23–25]. However, the incidence of THAs performed in ONFH has increased dramatically according to the analysis of an USA nationwide sample performed by Mont et al.: the proportion of ONFH treated with THA increased from 75% in 1992 to 88% in 2008. On the other hand, the proportion of joint-preserving procedures decreased from 25% to 12% [44]. Moreover, recent literature suggests a marked improvement in the survivorship and outcomes of THA when performed in ONFH population [27, 45–47]. There are only few studies presenting the clinical outcome of short stem THAs in ONFH at mid-term follow up. We therefore evaluated a series of patients 60 years old or younger who received a partial neck-retaining femoral short-stem with ceramic - on - ceramic bearings to determine mid-term clinical and functional results using validated scoring instruments.

The mid – term results in our series showed good to excellent clinical outcome and pain relief. At latest follow-up, mean HHS, WOMAC and UCLA activity scores were 90 (range, 71–100), 94 (range, 76–100), and 6.3 (range, 4–10) points(*p* < 0.001), respectively. Furthermore, there were no radiographic evidence of osteolysis and no need for revision. Our results are similar to the majority of reports by other researchers using cementless short-stem THAs in ONFH [16, 18, 48–51] (Table 4).

**Table 4:** Summary of studies regarding the outcome of partial neck-retaining short-stem total hip arthroplasty in patients with osteonecrosis of the femoral head

Study	Implant name	Implant design	Number of THAs	Bearings	Mean age (years)	Mean followup (years)	Mean Postoperative HHS	Stem revisions for aseptic loosening
Floerkemeier et al. [18]	Metha (B. Braun Aesculap)	Partial collum with neck preserving osteotomy	73	Poly – Ce Ce - Ce	49.4	3	90.6	0
Jerosch et al. [48]	Mini hip (Corin)	Partial collum with neck preserving osteotomy	20	Ce - Ce	36.2	4	HOOS: 93.9	0
Kim et al. [49]	Proxima (Depuy)	Wedge femoral neck-sparing short stem	144 (88 THAs in ONFH patients)	Ce - Ce	53.9	4.5	96	0
Wang et al. [51]	CFP (Link)	Modular femoral neck-sparing short stem	9	N/A	24.1	1.5	92.8	0
Zeh et al. [16]	Mayo (Zimmer)	Double-tapered short stem modular neck	26	N/A	44.9	7.9	93.5	0
Suksathien et al. [50]	Metha (B. Braun Aesculap)	Partial collum with neck preserving osteotomy	120	N/A	44.4	2.4	97.7	0
Current study	NANOS (Smith & Nephew)	Partial collum with neck preserving osteotomy	37	Ce - Ce	51.5	5.6	90	0

Floerkemeier et al. reported data of the Metha short-stem arthroplasty [18] in 73 patients who suffered from secondary osteoarthritis due to ONFH. At 3 years mean HHS score was 90,4 and no complication occurred during the follow-up. Zeh et al. [16] compared the midterm results of the MAYO short-stem THA in ONFH and in primary coxarthritis. After implantation of 26 Mayo short stem THAs in 21 patients, in the study group the postoperative HHS was 93.5 compared to 94.2 in the control group at 7.9 years mean follow - up. Recently, Jerosch et al. [48] reviewed the results of the MiniHip short-stem arthroplasty in 18 osteonecrotic hips. Hip Dysfunction Osteoarthritis and Outcome Score (HOOS) improved from 44.4 to 96.2 points at 4-year mean follow - up.

Moreover, our results are consistent even with clinical and radiological outcomes of standard stems arthroplasties due to ONFH [52, 53]. In a recent study, with mean follow-up time comparable to our series, Gao et al. [52] recruited 21 patients, 6 with bilateral necrosis (27 hips). A cementless standard stem was used in all hips and they reported a final follow-up mean HHS of 88.6 (*p* < 0.001). Cheung et al. [53] reported long-term results of a hydroxyapatite coated cementless femoral stem used in 117 total hip arthroplasties due to ONFH. At a mean follow-up of 14.7 years, HHS improved from mean preoperative 35.6 points to mean postoperative 83.8 (*p* < 0.001). Furthermore, they compared these results with a non-.

ONFH patient group of 65 hips and they found no statistically significant difference (*p* = 0.347).

Third generation ceramic bearings, according to published data allow low wear rates with the supposed benefit of remarkable long term survival even in young and active patients [54, 55]. Our results were comparable to those in other reports of THA using third-generation ceramic bearings in patients with osteonecrosis of the femoral head [56–61] (Table 5).

**Table 5:** Summary of studies regarding the outcome of ceramic bearings in patients with osteonecrosis of the femoral head

Study	Number of THAs	Mean age (years)	Mean follow up (years)	Head size	Mean postoperative HHS	Complications	Survivorship (endpoint)
Lim et al. [59]	53	49	5.3	32 mm in 11 hips 36 mm in 42 hips	97	noises 2/53 (4%) 1 squeaking 1 clicking	100% (revision)
Kim et al. [58]	93	38.2	11.1	28 mm	96	squeaking 2/93 (2%) recurrent dislocation 1/93 (1%) isolated dislocation 1/93 (1%)	99% (revision)
Millar et al. [60]	24	46	2,8	N/A	85.7	isolated dislocation 1/24 (1%)	N/A
Solarino et al. [61]	68	50	13	32 mm	90.7	N/A	95% (revision)
Evangelista et al. [57]	53	31	5.3	N/A	89	squeaking 3/53 (7%)	96.2% (revision)
Byun et al. [56]	56	25.6	7.7	N/A	98.2	squeaking 1/56 (2.4%)	100% (revision)

*N/A* not available

In our study, we did not found any evidence of squeaking and detected only one case (2.7%) in which occurred a clicking sound, tolerated by the patient, without sign of liner breaking or gross wear. As determined by previous studies [62–64], we speculated that patients who received a 36 mm head should have better function than 32 mm-head patients group. Thus, in our series the differences in functional scores between the two groups were not statistically relevant. Even more, we had only one case of early dislocation which has presented in a 36-mm head bearing. Similarly, Allen et al. [65] stated their study failed to show that increasing femoral head size significantly improves function 1 year after total hip arthroplasty, but showed that the use of a 36 mm or greater femoral head did reduce the dislocation rate. Lu et al. [66] found similar dislocation rates but better flexion in ≥36 mm group than in <36 mm group at 1–3 year after surgery.

The main concern of the opponents of short-stems use in ONFH is the theoretical increased risk of subsidence due to poor bone quality of the proximal femur [16, 67]. However, the results of the 37 hips evaluated in this study suggest that the concerns about poor secondary bone ingrowth and potential early revision of short-stems are possibly unfounded. In our series, osseointegration was seen in all hips and no signs of stem migration and osteolysis were detected at plain radiographs analysis in any of the 37 hips studied after a mean follow-up of 5.6 years. Kaipel et al. [68] assessed migration data in 49 NANOS short-stem arthroplasties, performed in patients affected by coxarthritis and ONFH, using a computer-assisted system. At 2-years follow-up, five (10%) stems showed vertical migration of more than 1.5 mm, but just in one case, distinctive subsidence could be monitored with conventional X-rays and was probably caused by under-sizing of the femoral implant. All other cases showing vertical migration at the software analysis had no correlation at the conventional X-rays and in clinical outcome.

This study has a number of limitations. Firstly, this is a single surgeon’s case series and there is no control group. The second limitation, is the length of the enrollment period and the small number of patients observed in the study. Thirty - seven hips, for thirty study patients with a minimum follow-up of three years are sufficient to detect early stem migration but does not allow analyzing influencing factors for implant failure such as gender, age, or implant size. Lastly, we did not use radiostereometric analysis to evaluate for migration, and this could have led to a lack of accuracy in radiographic measurements due to manual techniques.

## Conclusions

In summary, this study shows beneficial radiological data and excellent clinical mid-term results after the implantation of a partial neck-retaining short-stem with ceramic-on-ceramic bearings in ONFH patients. Based on the results of the present study, the migration and fixation pattern at mean 5.6-years follow-up predicts that a partial-neck retaining short stem could be a reliable option in advanced stage of ONFH either. Further investigations are crucial to determine the long-term durability of short stem THAs and to assess whether the association of ceramic-on-ceramic bearings, can be useful to achieve longer survivorship of the implants, better functional results and lower complications rates.

## Abbreviations

AP: anterior-posterior
Ce: Ceramic
HHS: Harris Hip Score
LLD: Leg length discrepancy
ONFH: Osteonecrosis of the femoral head
Poly: Polyethylene
THA: Total hip arthroplasty
UCLA: University of California, Los Angelese
WOMAC: Western Ontario and McMaster Universities Osteoarthritis Index
